# Perspectives on Li and transition metal fluoride phosphates as cathode materials for a new generation of Li-ion batteries

**DOI:** 10.1107/S205225251402329X

**Published:** 2015-01-01

**Authors:** Evgeny V. Antipov, Nellie R. Khasanova, Stanislav S. Fedotov

**Affiliations:** a Department of Chemistry, Lomonosov Moscow State University, Moscow 119991, Russian Federation

**Keywords:** Li-ion batteries, cathode materials, transition metal fluoride phosphates, structure–property relationships

## Abstract

To satisfy the needs of rapidly growing applications, Li-ion batteries require further significant improvements of their key properties: specific energy and power, cyclability, safety and costs. The first generation of cathode materials for Li-ion batteries based on mixed oxides with either spinel or rock-salt derivatives has already been widely commercialized, but the potential to improve the performance of these materials further is almost exhausted. Li and transition metal inorganic compounds containing different polyanions are now considered as the most promising cathode materials for the next generation of Li-ion batteries. Further advances in cathode materials are considered to lie in combining different anions [such as (*X*O_4_)^*n*−^ and F^−^] in the anion sublattice, which is expected to enhance the specific energy and power of these materials. This review focuses on recent advances related to the new class of cathode materials for Li-ion batteries containing phosphate and fluoride anions. Special attention is given to their crystal structures and the relationships between structure and properties, which are important for their possible practical applications.

## Abstract 2

The crystal structures of various mixed Li and transition metal fluoride phosphates are reviewed, with a special focus on their applicability as cathode materials for Li-ion batteries.

## 1. Introduction   

Today, 90% of the energy used in the world comes from fossil fuels, causing their rapid consumption followed by ecological damage and climate change. Together with the demand to increase the efficiency of using fossil fuels, there is a strong impetus to develop clean energy technologies, where new energy-storage devices will play an important role. Energy-storage devices allow energy buffering for peak use when power generation systems are operated at peak capacity. They are critical elements for solar, wind and water renewable energy technologies, mobile information and communications technologies, and hybrid/electric vehicles. In all these applications, electrochemical energy-storage devices have significant advantages over other competing technologies because they can offer much higher energy densities.

Li-ion batteries (LIBs) were originally developed for portable electronic devices, but nowadays new application niches are envisaged in electric vehicles and stationary energy storage. The rapid development of LIB production in the world during the last two decades was driven mainly by their gravimetric energy density (now exceeding 250 W h kg^−1^), which is several times higher than for alternative rechargeable battery systems. LIB systems also provide significant advantages in cyclability (the number of operating charge/discharge cycles), energy efficiency, absence of ‘memory’ effects, low discharge rate *etc*. The main disadvantages are relatively high costs and safety issues. To satisfy the needs of rapidly growing applications, Li-ion batteries require further significant improvements to their key properties: specific energy and power, cyclability, safety and costs.

The gravimetric energy density of an LIB is the product of its specific capacity (measured as the reversible charge transferred between the anode and cathode per unit weight) and the potential difference between these electrodes where reversible intercalation/deintercalation of Li ions occurs. The cathode is a key part of LIBs that significantly determines their performance. Severe requirements are imposed on the cathode material, which should demonstrate a high specific capacity, provide fast reversible intercalation of the Li ions at a redox potential close to the upper limit of the electrolyte stability window, possess a high electronic conductivity and a relatively low molecular weight, and exhibit a small variation in volume upon changing the Li concentration over a wide range. These properties are covered mainly by the crystal structure of the cathode material, and this is the reason for the large impact of crystallography research on the design of new cathode materials for LIBs and the optimization of their properties by appropriate modification of the chemical composition and, consequently, the crystal structure. It should be emphasized that the requirement for a high specific capacity (*C*_t_) of the cathode material restricts the number of elements that can be used due to the following equation: 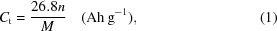
where *n* is the number of Li ions (and electrons) participating in the reversible redox reaction and *M* is the molecular weight of the material. By this equation, the selection of elements in the chemical formulae of Li-based compounds is limited to light transition metals such as Ti, V, Mn, Fe, Co and Ni. The requirement for fast Li-ion diffusion in the crystal structure of the cathode material (as it is a rate-limiting step of the redox process) is the key to achieving high-power LIBs because the diffusion time in a crystallite of the material is proportional to *R* ^2^/*D*, where *R* is the crystallite size and *D* is the diffusion coefficient.

Goodenough and co-workers explained, in a paper published in 1980 (Mizushima *et al.*, 1980 ▶), the selection of LiCoO_2_ as a possible cathode material with high specific energy because it possesses a suitable type of crystal structure. This α-NaFeO_2_-type structure is a layered derivative of the rock-salt structure, with a complete ordering of the Li and Co cations in the octahedral cavities between alternating close-packed oxygen layers. It is relatively light-weight, has a high average potential for Li-ion extraction (and Co^3+^/Co^4+^ transition) (close to 4 V *versus* Li^+^/Li^0^) and contains empty tetrahedral voids linked by common faces to the LiO_6_ octahedra that provide fast Li-ion two-dimensional diffusion, making this material attractive for LIBs. Ten years later, this discovery gave rise to the LIB industry, which nowadays requires further improvement in the cathode material properties for lower-cost longer-life higher energy/power density batteries, thus resulting in active development and research in this field. Since Goodenough’s pioneering work, various classes of cathode materials have been discovered, such as compounds based on the NASICON-like crystal structure [Li_3_Fe_2_(PO_4_)_3_], spinel LiMn_2_O_4_ with a three-dimensional pathway for Li-ion diffusion, and olivine LiFePO_4_ with a one-dimensional pathway, to name just a few.

The investigation of the NASICON-like compounds Li_3+*x*_Fe_2_(*X*O_4_)_3_ (*X* = P, S, Mo and W) performed by Goodenough and co-workers in the late 1980s allowed the formulation of the inductive effect concept, which explains the variation in electrode potential for these materials with the same structure type and close Fe—O bond distances by the changing electronegativity of the tetrahedrally coordinated *X* cation. This finding was an important step in postulating the ‘structure–property’ relationship for these cathode materials that enabled further tuning of their properties. A variation in the electronegativity of the *X* cation causes weakening or strengthening of the covalency of the Fe—O bonds, thus changing the energy of the Fe^3+^/Fe^2+^ redox couple and the respective electrode potential. For these materials, the electrode potential increases from 2.8 to 3.6 V *versus* Li^+^/Li^0^ from the less electronegative P cation to the most electronegative S cation, due to the higher ionicity of the Fe—O bonds for the sulfate-based compound (Goodenough & Kim, 2010 ▶).

The first generation of cathode materials for LIBs, based on mixed oxides with either spinel (Li*M*_2_O_4_, *M* = transition metal) or rock-salt derivatives (Li*M*O_2_), has already been widely commercialized, but the potential to improve the performance of these materials further is almost exhausted. Li and transition metal inorganic compounds containing different polyanions (*X*_*m*_O_*n*_)^*p*−^ (*X* = B, P, S, Si) are now considered as the most promising cathode materials for the next generation of LIBs for electric vehicles and large storage applications because their frameworks can provide long-term structural stability, which is essential for cyclability and safety. The best example among these materials is LiFePO_4_, discovered in 1997, which has already been widely commercialized (Padhi, 1997 ▶). Further advances in cathode materials are anticipated to be achieved by combining different anions [such as (*X*O_4_)^*n*−^ and F^−^] in the anion sublattice, which is expected to enhance the specific energy and power of these materials. Various fluoride phosphates (often called fluorophosphates) and fluoride sulfates have recently been discovered, and some of them do indeed exhibit attractive electrochemical performance. A wide selection of various polyanion-based cathode materials is described in the comprehensive review by Masquelier & Croguennec (2013 ▶).

In this review, we will focus on recent advances in the new and relatively narrow class of cathode materials for LIBs containing phosphate and fluoride anions. Special attention will be paid to their crystal structures and the relationships between structure and properties, which are important for their possible practical applications.

## 2. LiFePO_4_: crystal structure and Li-ion diffusion pathway   

Earth-abundant, environmentally benign and safe lithium iron phosphate, LiFePO_4_, has now reached prominence as a commercialized cathode material in LIBs for hybrid vehicles and grid storage systems. Upon Li extraction this material exhibits a flat plateau around 3.4 V *versus* Li^+^/Li^0^, because of a two-phase redox reaction mechanism involving the formation of fully delithiated FePO_4_.

The crystal structure of olivine-type LiFePO_4_ can be described as a slightly distorted hexagonal close-packed (h.c.p.) oxygen array with an orthorhombic unit cell (space group *Pnma*). Within the h.c.p. oxygen framework, the Li and Fe cations are located on half of the octahedral sites and the P atoms are located on 1/8 of the tetrahedral sites. The corner-sharing FeO_6_ octahedra generate layers (perpendicular to the *a* axis) that are bridged by the corner- and edge-sharing PO_4_ tetrahedra to form the structure framework with strong three-dimensional bonding. The LiO_6_ octahedra share edges and create linear chains running along the *b* axis.

It is fascinating how state-of-the-art technology has converted an insulator-type compound with poor electronic conductivity into a high-rate charge–discharge nanostructured electrode material, which delivers a specific capacity close to its theoretical value (170 mA h g^−1^) and exhibits a relatively high energy density of 583 W h kg^−1^. To achieve this electrochemical performance, many studies of the kinetics of the material and its intrinsic Li-ion mobility have been performed, including those using modern computational and first-principles methods such as density functional theory (DFT) and molecular dynamics (MD).

**Figure 1:**
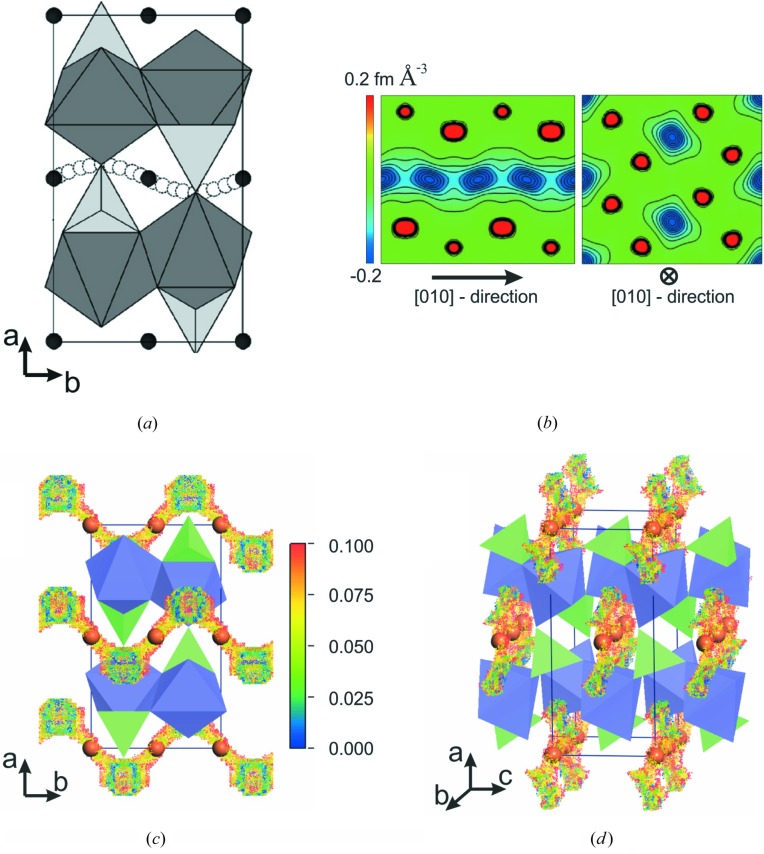
Visualizations of the curved one-dimensional Li-ion diffusion pathway in the LiFePO_4_ crystal structure by various methods. (*a*) From Islam’s work based on the MD approach. Adapted with permission from Islam *et al.* (2005 ▶). Copyright (2005) American Chemical Society. (*b*) The nuclear distribution of Li atoms given in the [010] and [001] directions, respectively. Adapted by permission from Nishimura *et al.* (2008 ▶). Copyright (2008) Macmillan Publishers Ltd, *Nature Materials*. (*c*) LiFePO_4_ BVS map sliced along the *ab* plane. (*d*) The crystal structure of LiFePO_4_ with an incorporated BVS map. Li ions are depicted as orange spheres, and blue octahedra and green tetrahedra designate FeO_6_ and PO_4_ polyhedra, respectively.

In 2005, Islam and co-workers demonstrated for the first time an application of the MD approach to the examination of Li^+^-ion diffusion pathways in LiFePO_4_ (Islam *et al.*, 2005 ▶). Despite the presence of two crystallographically possible channels for ion migration in the structure (along the *b* and *c* axes), the calculated activation barriers for Li-ion hopping confirmed the preferential accessibility of only the [010] direction, which proved the concept of one-dimensional transport for this material (Fig. 1 ▶ *a*). It was shown that Li^+^ ions follow a curved pathway, jumping through adjacent tetrahedral and octahedral voids, with an energy barrier of 0.55 eV. These results are in good agreement with the DFT calculations performed by Morgan *et al.* (2004 ▶).

At that time, the study of Li-ion transport was limited to computational predictions, and no experimental evidence of the diffusion pattern had been obtained until Nishimura *et al.* (2008 ▶) clearly visualized the migration pathway by means of high-temperature powder neutron diffraction and the maximum entropy method (MEM) (Fig. 1 ▶ *b*). The refined anisotropic atomic displacement parameters for Li ions at room temperature provided significant information on the overall anisotropy of the thermal vibration according to the shape of the ellipsoid. The thermal displacement is preferentially oriented towards adjacent face-sharing vacant tetrahedral voids, which produces the expected wave-like one-dimensional chain of Li^+^ ions.

The next experimental stage was to show the conversion of Li^+^-ion vibrations to actual diffusion. With this aim, the Li_0.6_FePO_4_ solid-solution composition was obtained at elevated temperature (above 570 K) with a significant number of lithium defects. Conventional structure models that include harmonic vibration of a static atom could not be exploited to investigate the dynamic disorder of the Li ions in the partially delithiated phase. The application of the MEM allows one to describe the complicated electron density or nuclear distributions that are beyond the classical static structure model, and therefore to evaluate the dynamic disorder of the Li ions by estimating the neutron scattering length density distribution. In crystallography, the MEM approach was first introduced by Collins (1982 ▶) and then validated for various materials with high ionic conductivity (Yashima *et al.*, 2003 ▶, 2005 ▶).

The resulting contour maps of the nuclear distribution of Li atoms sliced along the [010] and [001] directions are given in Fig. 1 ▶(*b*). The curved one-dimensional pathway along the [010] direction was successfully observed, providing long-expected experimental proof of the Li diffusion pathway in Li_*x*_FePO_4_. These findings are also consistent with the computational examinations given by Islam *et al.* (2005 ▶) and Morgan *et al.* (2004 ▶).

Both the above-mentioned theoretical and experimental approaches for probing Li-ion diffusion in similar materials are time-consuming, difficult to execute and sometimes require expensive preparations. In this case, a scientist should carefully choose the objects for these tests. That is why Li-ion migration is thoroughly examined by these approaches mainly in ‘renowned’ materials such as LiFePO_4_. However, there are many alternative electrode materials that are of interest for their Li-ion transport maps or overall kinetics. This fact facilitates the development of quick and simple tools for the preliminary investigation or prediction of ion-diffusion pathways in a material’s crystal structure.

Bond valence (BV) calculations were at first recognized as a rapid and efficient approach for validating the crystal structures of inorganic compounds (Brown, 2009 ▶). Later on, the method found some new applications. In particular, spatial distributions of BV values have been demonstrated to yield information concerning the topology of ion-conduction pathways (Adams, 2012 ▶; Adams & Rao, 2014 ▶). With this method to hand, one can not only draw tentative conclusions on the character of ion diffusion, but also significantly reduce the time and costs required for further examination by computational or complicated experimental techniques.

The BV sum (BVS) landscape for LiFePO_4_ perfectly reproduces the curved-shaped distribution of Li ions along the [010] direction. In Figs. 1 ▶(*c*) and 1 ▶(*d*), one may observe a zigzag pathway running along the *b* axis and passing through neighbouring occupied octahedral 4*a* sites and vacant tetrahedral sites. The intermediate tetrahedral positions for Li-ion hopping show very low BVS mismatch values. In other words, these sites are demonstrated to have a reasonably high probability of participating in Li-ion diffusion. To sum up, the BVS data accurately correlate with computational and experimental methods, which gives us an opportunity to use BVS calculations for preliminary prediction and characterization of the Li migration pathways in the cathode materials for LIBs that are discussed below.

In this paper we present BVS calculations for a set of cathode materials for LIBs. The calculations were performed using the *3DBVSMAPPER* program (Sale & Avdeev, 2012 ▶). The set of materials comprises LiFePO_4_, LiVPO_4_F with the tavorite structure type, and fluoride phosphates with the general formula Li_2_ *M*PO_4_F (*M* = Mn, Fe, Co), adopting three different types of framework.

## 3. Li and transition metal fluoride phosphates: crystal structure and Li-ion diffusion pathway   

Further advances in polyanion cathode materials are related to joining different units in the anion sublattice, which gives opportunities for varying the chemical composition and structural framework of the compounds and to tailor their electrochemical properties. Fluorine and oxygen, which are similar in size, can easily substitute each other in the anion sublattice. This combination of (*X*O_*n*_)^*p*−^ and F^−^ anions is expected to increase the operating voltage due to the higher ionicity of the *M*—F bond, which would ultimately provide a higher specific energy density. The difference in the formal charges of O^2−^ and F^−^ is expected to weaken Li bonding to the structure framework and enhance Li-ion transport, thus improving the power-density parameters. Bearing these considerations in mind, chemists have attempted to synthesize various fluoride-based polyanion cathode materials.

### 3.1. Fluoride phosphates with the tavorite structure type   

**Figure 2:**
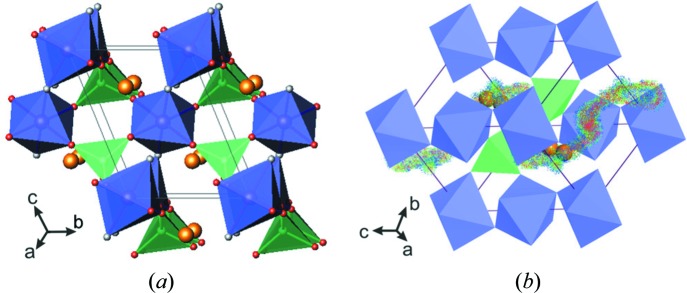
Representations of the LiVPO4F phase. (*a*) The crystal structure. (*b*) A BVS map of the Li^+^-ion transport pathway. Transition metal octahedra are shown in blue, phosphate tetrahedra in green, F in grey and Li ions in orange.

The first fluoride-based polyanion cathode material was introduced by Barker *et al.* (2003 ▶), who reported Li-ion insertion/extraction activity in the fluoride phosphate phase LiVPO_4_F, prepared by the carbothermal reduction method. LiVPO_4_F is isostructural with the mineral tavorite, LiFePO_4_OH, and crystallizes in a triclinic unit cell (space group 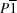, *Z* = 2) with *a* = 5.1688 (2) Å, *b* = 5.3094 (2) Å, *c* = 7.4994 (2) Å, α = 113.12 (1)°, β = 112.93 (2)°, γ = 81.66 (2)° and *V* = 174.3 Å^3^. Its crystal structure is built up of two slightly distorted crystallographically independent (VO_4_F_2_) octahedra bridged by fluorine vertices in the *trans*-positions, to form one-dimensional chains along the *c* direction. These (VO_4_F_2_)_∞_ chains are interconnected through phosphate groups and this packing creates a three-dimensional framework with wide tunnels that host the Li ions (Fig. 2 ▶ *a*). According to Ellis *et al.* (2011 ▶), the Li ions are randomly distributed over two crystallographic positions separated by 0.79 Å: the five-coordinated Li1 and six-coordinated Li2 sites. The occupancies of the Li1 and Li2 sites were found to be 0.18 and 0.82, respectively, and this finding was confirmed by NMR measurements. From combined X-ray and neutron powder diffraction data, Ateba Mba *et al.* (2012 ▶) suggested an occupancy for the single Li site corresponding to the major occupied position reported by Ellis and co-workers.

The structural framework of LiVPO_4_F implies four crystallographically distinct channels (along the [100], [010], [101] and [111] directions) suitable for Li-ion migration. BVS calculations for this material revealed a very complicated diffusion pathway for the Li ions. As shown in Fig. 2 ▶(*b*), it passes preferentially along the [111] direction. This finding is in good agreement with the work of Mueller *et al.* (2011 ▶) claiming that this direction, with the lowest energy barrier of 208 meV, is the most favorable for Li-ion migration.

The electrochemical activity of LiVPO_4_F is a good example representing the inductive effect caused by fluorine. The potential of Li deintercalation involving the V^4+^/V^3+^ redox couple is near 4.2 V, *i.e.* about 0.5 V higher than the potential for the same transition observed in Li_3_V_2_(PO_4_)_3_ with the NASICON structure (Gaubicher *et al.*, 2000 ▶), whereas the observed capacity value of 155 mA h g^−1^ corresponds to the reversible uptake of 0.97 Li ions (Barker *et al.*, 2003 ▶). Moreover, this system exhibits the possibility of realising multi-electron redox activity based on the multivalent nature of vanadium: the reversible insertion of 0.9 Li ions (involving the V^4+^/V^3+^ redox transition) takes place at ∼1.8 V and corresponds to a capacity value of 140 mA h g^−1^ (Barker *et al.*, 2005 ▶). Both redox reactions were shown to be covered by a two-phase mechanism and accompanied by significant volume changes: about 8.5% for the deintercalation reaction (LiVPO_4_F → VPO_4_F) and 7.4% for the LiVPO_4_F → Li_2_VPO_4_F transition (Ellis *et al.*, 2011 ▶). Although the total capacity value of Li_1+*x*_VPO_4_F exceeds 290 mA h g^−1^, the large difference between these two redox reactions (2.4 V) makes the application of this system in its multivalent form problematic. An application of this material in a symmetrical cell at both positive and negative electrodes was proposed by Barker (2005 ▶), but it exhibited poor cycling stability due to dissolution of LiVPO_4_F at the anodic site in the acidic LiPF_6_-based organic electrolyte. Nevertheless, switching to an ionic liquid electrolyte provided a much more stable and highly reversible performance of the symmetrical cell at room and elevated temperatures (Plashnitsa *et al.*, 2011 ▶).

Despite the attractive electrochemical properties demonstrated by the Li_1+*x*_VPO_4_F system, the high cost and toxicity of vanadium compounds inspired a search for new fluoride phosphate cathode materials among other 3*d* transition metals. Lithium iron fluoride phosphate, LiFePO_4_F, iso­structural with tavorite, was prepared by different synthetic routes, including solid-state reaction and ionothermal or solvothermal synthesis (Recham *et al.*, 2010 ▶; Ramesh *et al.*, 2010 ▶; Ellis *et al.*, 2012 ▶). It crystallizes in the triclinic space group 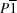 with cell parameters *a* = 5.1516 (2) Å, *b* = 5.3002 (2) Å, *c* = 7.2601 (2) Å, α = 107.343 (3)°, β = 107.880 (3)°, γ = 98.559 (3)° and *V* = 173.67 (6) Å^3^. According to Rietveld refinement of powder X-ray diffraction data, the Li ions are located on two independent crystallographic sites, Li1 and Li2, with partial occupancies of 0.75 and 0.25, respectively (Ellis *et al.*, 2012 ▶).

In contrast with LiVPO_4_F, lithium extraction from LiFePO_4_F was not observed since it would involve the Fe^3+^/Fe^4+^ redox reaction taking place at very high potentials. LiFePO_4_F is capable of reversible Li intercalation, with an average redox potential of 2.8 V and a reversible capacity of 145 mA h g^−1^, close to the theoretical value (146 mA h g^−1^). Li insertion results in considerable volume expansion (∼8%) and gives the new Li_2_FePO_4_F phase. Its structure was refined in the space group 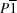 [*a* = 5.3276 (2) Å, *b* = 5.3736 (2) Å, *c* = 7.4791 (2) Å, α = 108.271 (4)°, β = 108.398 (4)°, γ = 94.615 (4)°) and *V* = 189.03 (4) Å^3^] on the basis of combined X-ray and neutron powder diffraction data. It was found that Li insertion keeps intact the corner-shared ‘FePO_4_F’ framework of the parent tavorite structure and leads to the appearance of two additional sites for the Li ions. According to electrochemical and X-ray diffraction data, Li intercalation in LiFePO_4_F is rather complex, as it involves occupation of the first Li site *via* a solid-solution (single-phase) process, followed by occupation of the second Li site through a two-phase mechanism (Ellis *et al.*, 2012 ▶). LiFePO_4_F exhibits excellent electrochemical performance with low polarization and good cycling retention (Ramesh *et al.*, 2010 ▶; Ellis *et al.*, 2012 ▶). However, the low redox voltage makes its applications questionable, due to its low energy density, and limited to batteries with a metallic Li anode (Li metal batteries).

### 3.2. Fluoride phosphates with general formula *A*_2_ *M*PO_4_F   

These compounds with *A* = Li, Na and *M* = Fe, Mn, Co and Ni have received particular interest due to their potential to operate on more than one alkali atom per transition metal, which would result in high specific capacities (>200 mA h g^−1^). Depending on the nature of the alkali metals, the 3*d* transition metals and the synthesis conditions, these fluoride phosphates adopt three structure types. In all three types, transition metals occupy the octahedral sites, but the connectivity of the *M*O_4_F_2_ octahedra is different, varying from mixed face-shared and corner-shared in the layered structure [(Na,Li)_2_ *M*PO_4_F with *M* = Fe, Co, Ni; Ellis *et al.*, 2010 ▶] to edge-shared in the three-dimensional orthorhombic structure (Li_2_ *M*PO_4_F, *M* = Co, Ni; Dutreilh *et al.*, 1999 ▶; Okada *et al.*, 2005 ▶) and corner-shared in the three-dimensional monoclinic structure (Na_2_MnPO_4_F; Yakubovich *et al.*, 1997 ▶).

#### 3.2.1. The crystal structures of Na_2_ *M*PO_4_F (*M* = Fe, Co, Ni)   

**Figure 3:**
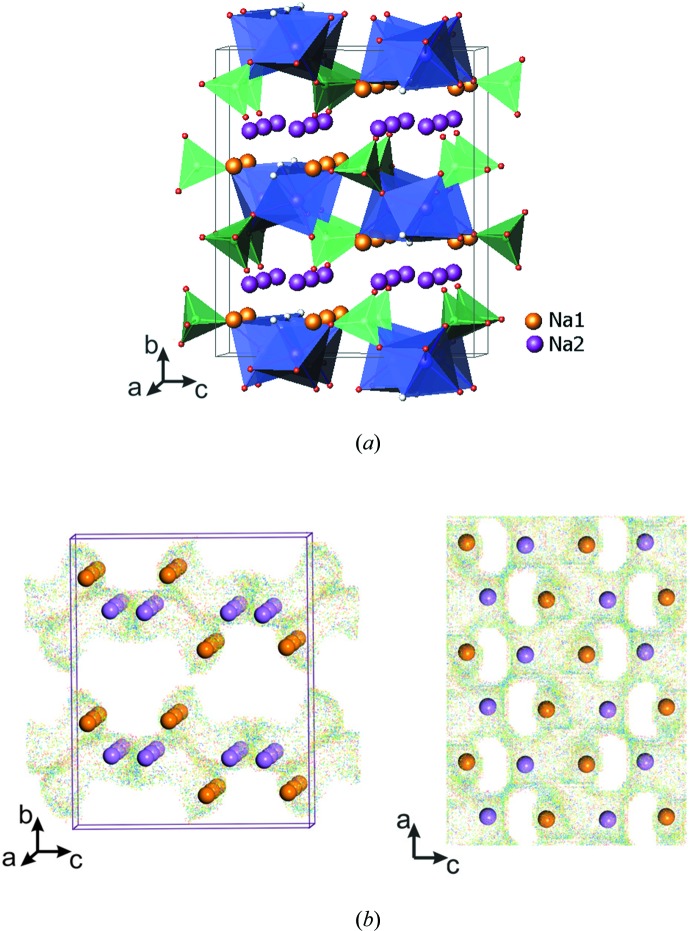
Representations of the Na_2_MPO_4_F fluoride phosphate. (*a*) The crystal structure. (*b*) Projections of BVS maps of alkali-ion migration pathways in the (011) and (101) layers. The *M*O_4_F_2_ octahedra are depicted in blue, phosphate tetrahedra in green, F in grey, and alkali ions in purple and orange.

They consist of a layered framework described in the orthorhombic space group *Pbcn*. Bi-octahedral *M*_2_O_7_F_2_ units comprising face-sharing *M*O_4_F_2_ octahedra are connected *via* bridging F atoms to form chains, and these are interconnected by PO_4_ tetrahedra to yield [*M*PO_4_F] infinite slabs. The Na atoms occupy two distinct crystallographic sites located in the interlayer space and possess facile two-dimensional migration pathways (Fig. 3 ▶ *a*). Although this type of fluoride phosphate has been stabilized for different transition metals (*M* = Fe, Co, Ni), detailed investigation of their structures and electrochemical properties was only carried out for the iron-based fluoride phosphate Na_2_FePO_4_F [*a* = 5.2200 (2) Å, *b* = 13.8540 (6) Å, *c* = 11.7792 (5) Å and *V* = 851.85 Å^3^; Ellis *et al.*, 2007 ▶].

Na_2_FePO_4_F and corresponding electrode materials were obtained by various synthetic routes (solid-state synthesis, sol–gel technique, and hydrothermal and ionothermal methods) which enable the preparation of materials with different morphologies and particle sizes (Ellis *et al.*, 2007 ▶; Recham *et al.*, 2010 ▶). Electrochemical testing of Na_2_FePO_4_F in an Li cell revealed reversible electrochemical activity with an average potential of 3.3 V (*versus* Li^+^/Li^0^). It delivered a specific capacity of 115 mA h g^−1^ (85% of the theoretical value) and showed good cycling sustainability. These observations confirmed the possibility of sodium-containing materials cycling reversibly in lithium-based electrolytes: as Na^+^ ions have been extracted from the framework upon electrochemical oxidation, Li^+^ ions become intercalated into NaFePO_4_F, resulting in a compositional transformation to (Na,Li)_2_FePO_4_F, which continues to function as an Li cathode upon subsequent cycling. This new material exhibited a sloping voltage profile, suggesting a quasi-solid solution electro­chemical behaviour, with a volume contraction of 3.7%, which is much smaller than that observed for LiFePO_4_. The electrochemical activity of both Na_2_FePO_4_F and (Na,Li)_2_FePO_4_F involves only one electron redox transition, attributed to the Fe^2+^→Fe^3+^ reaction, with a reversible extraction of one alkali ion. By an ion-exchange reaction, Na was completely substituted by Li to result in a new Li_2_FePO_4_F phase with a layered two-dimensional structure (*a* = 5.0550 Å, *b* = 13.5610 Å, *c* = 11.05200 Å, *V* = 757.62 Å^3^; Ellis *et al.*, 2007 ▶). BVS calculations (Fig. 3 ▶ *b*) confirmed the two-dimensional migration pathways realised in this framework. Therefore, in the Na_2_FePO_4_F structure, both alkali metal sites are open for facile ion transport, and the limitations observed upon electrochemical deintercalation seem to be caused by the high potential of the Fe^3+^→Fe^4+^ oxidation. Li_2_FePO_4_F showed a voltage charge–discharge profile very similar to that of Na_2_FePO_4_F after five cycles and delivered about 110 mA h g^−1^ capacity at an average potential of 3.3 V (Ellis *et al.*, 2007 ▶).

#### 3.2.2. Fluoride phosphate Na_2_MnPO_4_F   

**Figure 4:**
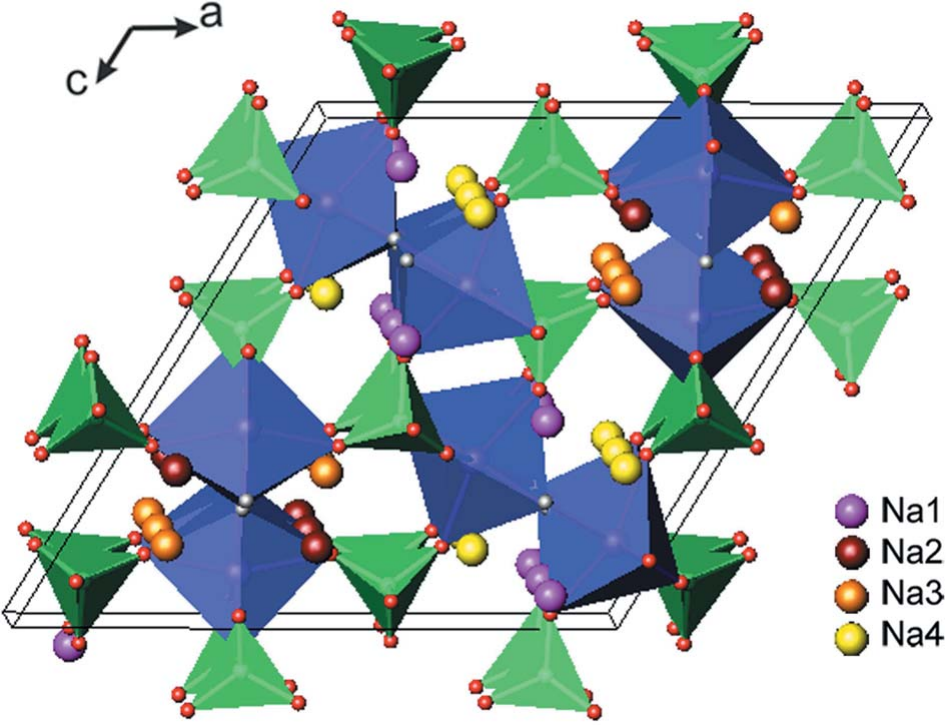
The crystal structure of the Na_2_MnPO_4_F fluoride phosphate. The MnO_4_F_2_ octahedra are shown in blue, phosphate tetrahedra in green and F in grey. The inset indicates the alkali metal positions.

This compound crystallizes in a three-dimensional monoclinic structure [space group *P*2_1_/*n*, with *a* = 13.683 (3) Å, *b* = 5.317 (1) Å, *c* = 13.711 (3) Å, β = 119.67 (3)° and *V* = 867.1 Å^3^; Yakubovich *et al.*, 1997 ▶]. In this structure, the alternate MnO_4_F_2_ octahedra are corner-shared and connected through fluorine to form Mn_2_O_8_F_2_ chains, with the F-ion backbone running along the *b* axis. These chains are linked by PO_4_ tetrahedra to form a three-dimensional framework, with Na^+^ ions located in the channels on four independent crystallographic sites (Fig. 4 ▶).

Despite an open pathway for alkali-ion migration, initial reports on Na_2_MnPO_4_F revealed the absence of any reversible electrochemical activity, which was explained by poor Na-ion diffusion kinetics (Recham *et al.*, 2009 ▶; Ellis *et al.*, 2010 ▶). Later on, Wu *et al.* (2011 ▶) prepared a carbon-coated nano-sized Na_2_MnPO_4_F material by a sol–gel technique and tested it in an Li cell. At elevated temperature (330 K) this material delivered a reversible capacity of 98 mA h g^−1^ (for the first discharge), which faded rapidly upon cycling. Kim *et al.* (2012 ▶) investigated the electrochemical properties of Na_2_MnPO_4_F prepared through a solid-state reaction and evaluated the diffusion kinetics of alkali ions using first-principles calculations. They showed that the *b*-direction diffusion of Na^+^ ions (along the F-ion backbone) is valid in Na_2_MnPO_4_F, in agreement with the reversible electrochemical performance of Na_2_MnPO_4_F in an Na cell: the observed discharge capacity of 120 mA h g^−1^ indicated the transfer of almost one Na ion. Furthermore, by an ion-exchange reaction they obtained a new Li_2_MnPO_4_F phase (isostructural with the parent phase), which demonstrated a discharge capacity of 140 mA h g^−1^ with a redox potential plateau at around 3.9 V (Kim *et al.*, 2012 ▶).

**Figure 5:**
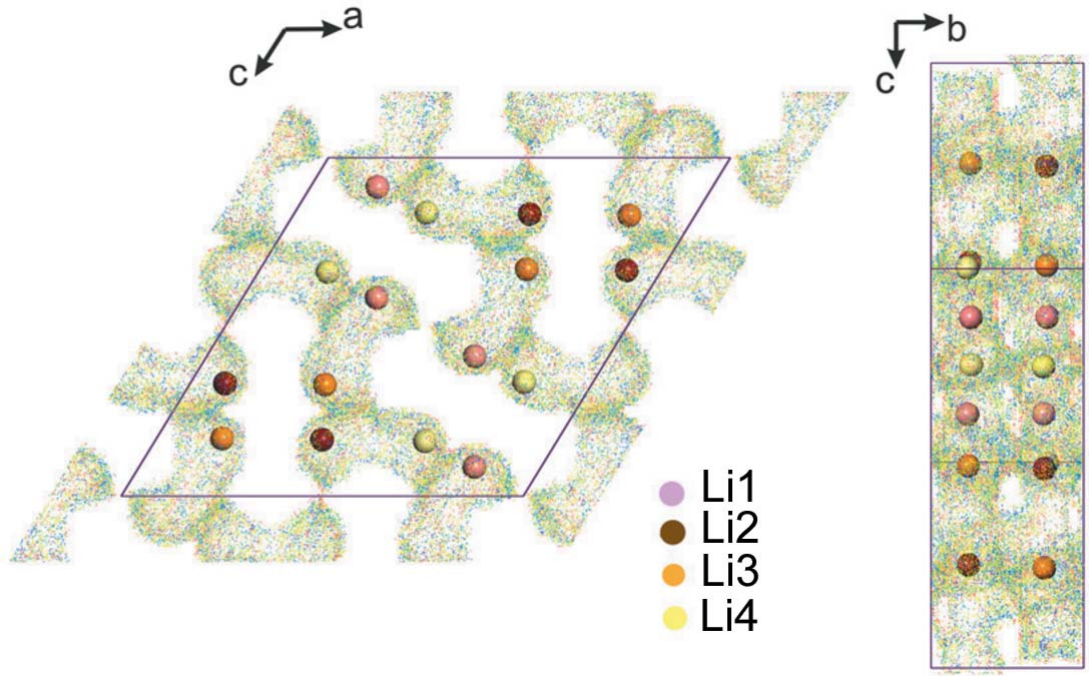
BVS mapping of Li^+^-ion transport pathways in Li_2_MnPO_4_F: projections in the (101) and (011) layers. The inset indicates the alkali metal positions.

Analysis of Li-ion transport in the new Li_2_MnPO_4_F phase revealed that, in addition to *b*-direction diffusion, the Li ions are able to migrate through pathways perpendicular to the F-ion backbone, which were forbidden for Na-ion diffusion because of the high activation energy (Kim *et al.*, 2012 ▶). These results are in a good agreement with the data of BVS mapping for Li_2_MnPO_4_F that clearly indicated two-dimensional migration pathways: along the *b* axis and along the diagonal of the *ac* plane (Fig. 5 ▶). These findings suggested that Li_2_MnPO_4_F would exhibit a more facile Li-ion diffusion.

For both of the manganese-based fluoride phosphates, Na_2_MnPO_4_F and Li_2_MnPO_4_F, electrochemical activity was limited to reversible transfer of about one alkali metal. Kim *et al.* (2012 ▶) calculated the average voltages for the first and second alkali metal extractions and showed that, with cut-off voltages above 4.8 V, more than one Na or Li ion would be extracted, but the electrolyte used could not sustain these high voltages.

#### 3.2.3. Fluoride phosphates, Li_2_ *M*PO_4_F (*M* = Ni, Co)   

**Figure 6:**
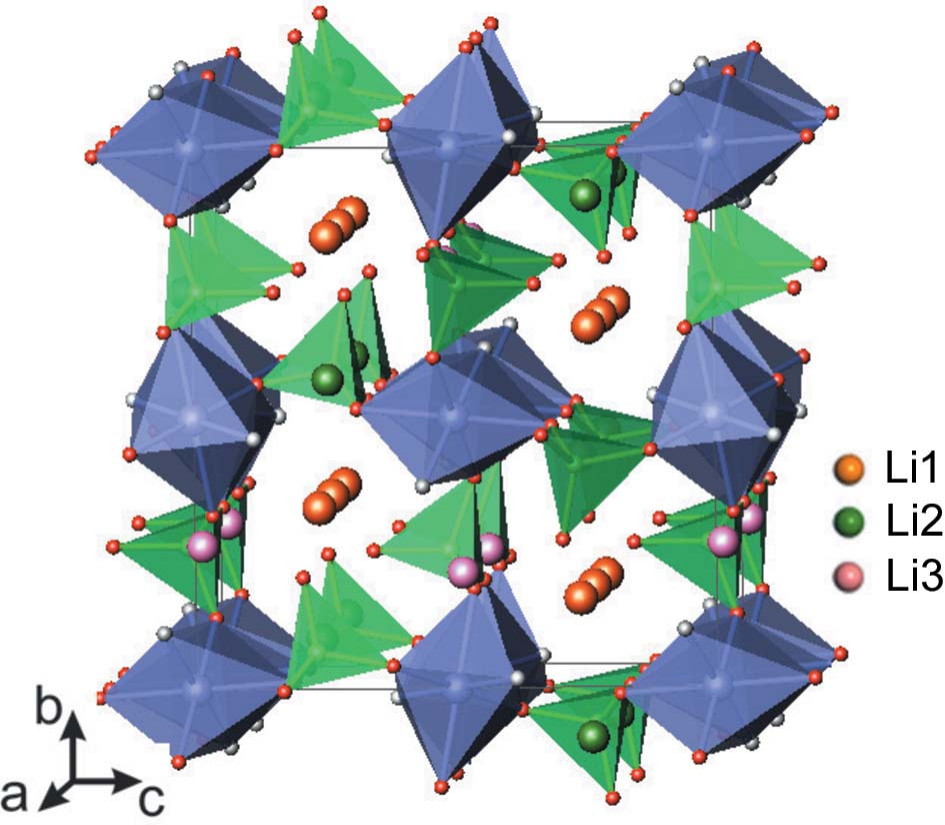
The crystal structure of the Li_2_CoPO_4_F fluoride phosphate. The CoO_4_F_2_ octahedra are shown in blue, phosphate tetrahedra in green and F in grey. Alkali metal positions are indicated in the inset.

**Figure 7:**
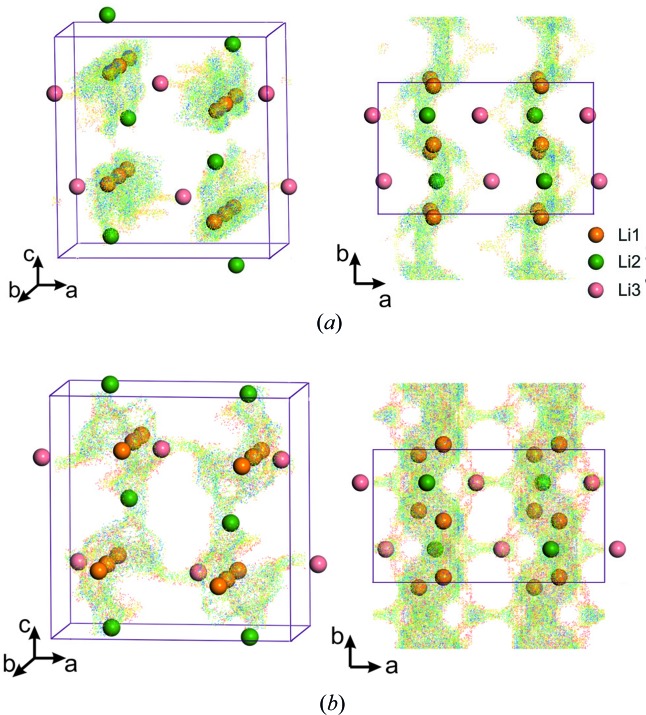
BVS maps of Li^+^-ion migration in (*a*) the Li_2_CoPO_4_F structure and (*b*) the chemically oxidized Li_1.3_CoPO_4_F phase. Projections in the (101) and (110) layers are given. The inset indicates the alkali metal positions.

These compounds, with an orthorhombic three-dimensional structure, can be prepared by direct synthesis. Li_2_NiPO_4_F was reported in 1999 (Dutreilh *et al.*, 1999 ▶), while Li_2_CoPO_4_F (space group *Pnma*, with *a* = 10.444 Å, *b* = 6.381 Å, *c* = 10.864 Å and *V* = 724.1 Å^3^) was introduced in 2005 by Okada, who proposed to use it as a high-voltage cathode material (Okada *et al.*, 2005 ▶). The structure type is built of *M*O_4_F_2_ octahedra linked through their edges to form rutile-like chains, with F atoms in *trans*-positions (Fig. 6 ▶). These parallel chains are interconnected through phosphate groups, and this packing creates a three-dimensional framework with large tunnels along the [010] direction which accommodate the Li ions in three distinct positions with full occupancy: two Li sites (Li1 and Li2) are five-coordinated, while the third one (Li3) has a distorted six-coordinated environment (Dutreilh *et al.*, 1999 ▶; Hadermann *et al.*, 2011 ▶). Analysis of the spatial distribution of BVS values using structure data obtained from combined neutron and X-ray diffraction data (Khasanova *et al.*, 2015 ▶) suggested one-dimensional Li-ion diffusion along the [010] direction with de/intercalation of one Li ion per formula unit from the ‘most open’ Li1 site, while participation of the Li2 site located on the isosurface edge was found to be questionable (Fig. 7 ▶ *a*).

Investigation of the electrochemical properties of these fluoride phosphates is hampered by their high redox potentials: ∼5 V for Li_2_CoPO_4_F and even higher (>5.3 V) for Li_2_NiPO_4_F (Okada *et al.*, 2005 ▶; Nagahama *et al.*, 2010 ▶). During the first charge, Li_2_CoPO_4_F was found to undergo an irreversible structural transformation involving mutual rotations of CoO_4_F_2_ octahedra and PO_4_ tetrahedral units with a 5% unit-cell expansion (Khasanova *et al.*, 2011 ▶). This expansion, which makes the framework ‘more open’, is expected to facilitate Li mobility upon subsequent cycling. This assumption was confirmed by the calculation of the spatial distribution of BVS values carried out for the chemically oxidized fluoride phosphate Li_1.3_CoPO_4_F [space group *Pnma*, with *a* = 10.9492 (9) Å, *b* = 6.2831 (8) Å, *c* = 11.680 (11) Å and *V* = 760.43 (1) Å^3^]. As can be seen from Fig. 7 ▶(*b*), this transformation resulted in considerable expansion of the diffusion channels along the *b* axis, with involvement of the Li1 and Li2 sites in the diffusion process, which would supply a higher specific capacity. Furthermore, this transformation seemed to provide an additional curved Li-migration pathway along the *c* axis, which would improve Li-ion transport (Khasanova *et al.*, 2015 ▶).

According to the capacity–voltage dependence obtained by potentiostatic step measurements, the extraction of more than one Li^+^ ion from Li_2_CoPO_4_F should take place at potentials >5.5 V, *i.e.* beyond the stability window of commercial electrolytes. Li_2_CoPO_4_F delivers a reversible discharge capacity of 60 mA h g^−1^ when the potential window is limited to 5.0 V *versus* Li^+^/Li^0^, and this value is increased up to 90 mA h g^−1^ when the upper potential limit is shifted to 5.5 V (Khasanova *et al.*, 2011 ▶). An initial discharge capacity of 130 mA h g^−1^ (corresponding to intercalation of ∼0.9 Li) was detected in a high-voltage electrolyte with fluorinated alkyl carbonates, but noticeable capacity fading was observed upon prolonged cycling (Amaresh *et al.*, 2012 ▶). Therefore, evaluation of the electrochemical performance of Li_2_CoPO_4_F and its practical potentials is restricted by the inaccessibility of stable high-voltage electrolytes.

Another way of exploring this fluoride phosphate system is to adjust (to decrease) the operating voltage of these compounds to values sustained by conventional electrolytes. This might be achieved through a complete or partial substitution of Co^2+^ by transition metals with lower values of *M* ^2+^/*M* ^3+^ redox potentials (Fe^2+^ or Mn^2+^). The Li_2_Co_1−*x*_Fe_*x*_PO_4_F and Li_2_Co_1−*x*_Mn_*x*_PO_4_F systems were found to exhibit very limited ranges of solid solution, with little effect on the redox potential value (Khasanova *et al.*, 2013 ▶). These results are explained by the large difference in the ionic sizes of these transition metals: the framework seems to become unstable upon higher substitution of Co^2+^ (ionic radius 0.735 Å) by larger Fe^2+^ (0.780 Å) and Mn^2+^ (0.820 Å) [ionic radii from Shannon (1976 ▶)]. Indeed, while Li_2_ *M*PO_4_F (*M* = Co, Ni) can be prepared by direct synthesis, the synthesis of three-dimensional Li_2_FePO_4_F requires electrochemical ion exchange using the Na counterpart, NaLiFePO_4_F, and the corresponding Mn-based fluoride phosphate has not yet been identified.

The NaLiFePO_4_F phase was obtained by solid-state synthesis, reacting an equimolar mixture of NaF and LiFePO_4_ (Khasanova *et al.*, 2012 ▶; Ben Yahia *et al.* 2012 ▶). It was found that, upon cycling in an Li cell, this compound undergoes a compositional transformation, giving a rise to a new polymorph of Li_2_FePO_4_F with a three-dimensional structure. This new phase exhibited a sloping voltage profile at an average potential of 3.4 V, delivering a reversible capacity of 113 mA h g^−1^ (∼0.84 Li) with a 1.7% volume change between charged and discharged states (Khasanova *et al.*, 2012 ▶). This good cycling performance, the small change in unit-cell volume and the solid-solution electrochemical behaviour, which implies the absence of serious kinetic limitations, make this fluoride phosphate system suitable for a reversible de-/intercalation of Li, especially if the energy density of this system can be enhanced.

## 4. Conclusions and future outlook   

Mixed Li and transition metal fluoride phosphates exhibit a rich crystal chemistry, owing to the variety of association modes of *M*O_*x*_F_*y*_ polyhedra (mainly *M*O_4_F_2_ octahedra) and PO_4_ tetrahedra in polyhedral networks of different dimensionality, thus providing a large playground for the design of new cathode materials for Li-ion batteries. Several types of such compounds, which are currently studied by different research groups, and their relevant electrochemical properties are listed in Table 1 ▶. This field of inorganic and materials chemistry is obviously very interesting for the academic community because it opens up great perspectives for the discovery of new structure types and unconventional synthesis routes. However, the practical application of these materials raises serious doubts when compared with LiFePO_4_ due to the following reasons:

(*a*) More complicated (and costly) synthesis techniques;

(*b*) Smaller (in general) gravimetric and volumetric energy densities for cheap Fe-based compounds if only one Li ion is extracted;

(*c*) Side-reactions with electrolyte and, consequently, a low Coulomb efficiency for high-voltage Co-based materials;

(*d*) A relatively large volume variation for V-based fluoride phosphates upon Li extraction or its dissolution in organic electrolytes for the heavily reduced phase.

Nevertheless, these compounds exhibit several advantageous properties that make them not simply ‘objects of scientific curiosity’ but provide some hopes for practical applications. The most important are the following:

(*a*) These phases may have a relatively high Li-ion diffusion coefficient, due to the greater free volume for Li-ion migration compared with close-packed mixed oxide structures, and weaker Li^+^—F^−^ bonding compared with Li^+^—O^2−^ ones. This feature is important for high-power batteries and their application at low temperatures.

(*b*) Several phases have high values of specific energy, significantly exceeding the value for LiFePO_4_. They can be used for batteries with high gravimetric energy densities, if the problem with high-voltage liquid electrolytes can be solved, or for applications in all solid-state ceramic batteries.

(*c*) Obviously, the extraction of more than one Li ion per formula will provide a significant increase in specific energy. However, the problem of a stable high-voltage electrolyte would be the ‘bottle-neck’, as in the previous case.

(*d*) Last, but not least, some of these phases are attractive for application in Na-ion batteries because they can be easily produced and Na ions exhibit high mobility, as shown by Li-based analogues obtained by ion-exchange chemically or electrochemically.

The rapid development of the rechargeable batteries industry requires the exploration of different types of material because their properties are the most important factor limiting progress in this field. The LIB is a complicated system combining different types of inorganic, organic and polymer materials. Mixed polyanion-based fluorides may open up new possibilities once the respective problems are solved for the whole set of materials to be used in LIBs.

## Figures and Tables

**Table 1:** Selected electrochemical properties of Li and transition metal fluoride phosphates

Chemical composition	Dimensionality of polyhedral network/Li-ion diffusion pathway	Average potential *versus* Li^+^/Li^0^ (V)	Theoretical specific capacity (mAhg^1^)/energy (Whkg^1^)†
LiFePO_4_	Three-dimensional/one-dimensional	3.43	170/583
LiVPO_4_F	Three-dimensional/one-dimensional	4.2	156/655
Li_2_VPO_4_F	Three-dimensional/one-dimensional	1.8	150/270
Li_2_FePO_4_F (tavorite type)	Three-dimensional/one-dimensional	2.9	146/423
Li_2_FePO_4_F (layered type)	Two-dimensional/two-dimensional	3.3	146/482
Li_2_FePO_4_F (three-dimensional type)	Three-dimensional/onetwo-dimensional	3.4	146/496
Li_2_MnPO_4_F	Three-dimensional/two-dimensional	3.9	147/573
Li_2_CoPO_4_F	Three-dimensional/onetwo-dimensional	5.1	143/730

† Calculated for extraction of one Li *versus* metallic Li anode.

## Appendix A BVS landscape computational procedure

The algorithm for calculating BVS mismatch maps is competently implemented in the *3DBVSMAPPER* program (Sale & Avdeev, 2012 ▶), which was utilized in this work. The program is capable of automatically producing a three-dimensional canvas of BV values for a given test ion (Li^+^), based on the following equation

where *d_j_* is the distance to the *j*th counter-ion site with occupancy *m*_*j*_, *R*_0_ and *b* are tabulated constants which are dependant on the type of Li^+^ ion and the *j*th neighbour, respectively, and *N* is the number of counter-ions within the present cut-off distance.

This formula does not consider Coulomb repulsion, but *3DBVSMAPPER* generates blocking spheres around atoms with the same sign of oxidation state as Li^+^ (in this work, P^5+^ and *M* ^*n*+^) to avoid building unphysical landscapes. Also, the initial position of Li^+^ ions is ignored when performing the BVS calculation to analyse the crystal structure for all possible ionic transport pathways.

Crystallographic information files (CIFs) for Li_2_FePO_4_F and Li_2_MnPO_4_F were artificially created by substitution of sodium by lithium in the original CIFs for the Na counterparts (Na_2_FePO_4_F and Na_2_MnPO_4_F, respectively; Ellis *et al.*, 2007 ▶; Yakubovich *et al.*, 1997 ▶) and shrinkage of the unit cell to the values refined from X-ray powder diffraction patterns. All the BVS landscapes within the scope of this article were calculated according to a ±0.1 deviation of BV value from the control value for an Li^+^ ion (which is equal to 1) with a fine spatial resolution of 0.2 Å. The magnitude of the BVS deviation is scaled in a reverse-rainbow manner, ranging from dark blue (0.0) to red (0.1) in both directions.
